# Fabry Disease Diagnosed With Lung Nodule: A Unique Presentation in a Young Female Patient

**DOI:** 10.7759/cureus.112962

**Published:** 2026-07-19

**Authors:** Joseph Joppich, Shriya Mehta, Nishanth Mandavilli, Frank Rojas Alvarez, Vikas Mehta

**Affiliations:** 1 Pathology, Northwestern University Feinberg School of Medicine, Chicago, USA; 2 Pathology, University of Michigan, Ann Arbor, USA; 3 Pathology, Northwestern Memorial Hospital, Chicago, USA

**Keywords:** benign lung nodule, fabry's disease, genetic syndromes, histiocytic, pseudo tumor

## Abstract

Fabry disease, also known as Anderson-Fabry disease, is a rare, X-linked lysosomal storage disease caused by mutations in the *GLA* (galactosidase alpha) gene, which causes a deficiency in the action of the alpha-galactosidase A enzyme. This often leads to an accumulation of globotriaosylceramide and globotriaosyl-sphingosine in various tissues, allowing clinical presentations of Fabry disease to be quite diverse among patients. This report demonstrates a case of a 31-year-old woman with a unique presentation of Fabry disease. The patient presented to the emergency room with two days of cough and hemoptysis. A CT scan revealed a 2.5 cm x 2.6 cm x 2.4 cm lung nodule in the right middle lobe. This nodule was eventually removed and sent for pathological examination, where it was determined to be a likely manifestation of Fabry disease. Genetic testing confirmed the diagnosis of a *GLA* c.988C>G, p. Q330E variant classified as likely pathogenic. Due to the absence of other signs or symptoms of Fabry disease after further workup, the patient was recommended for biannual follow-up. This case demonstrates a unique presentation of Fabry disease that may be helpful for clinicians when determining the etiology of lung nodules, especially in patients with a family history of the disease. Furthermore, recognition of nodule formation as a potential presentation of Fabry disease is important for effective diagnosis and long-term management of patients with the disease as a whole.

## Introduction

Fabry disease (FD) results in the complete absence or partial deficiency of the alpha-galactosidase A enzyme, leading to accumulation of various glycosphingolipids, including globotriaosylceramide (Gb3) and globotriaosyl-sphingosine (Lyso-Gb3), in both plasma and tissue lysosomes [1,2]. This lysosomal accumulation leads to progressive damage and dysfunction across multiple organ systems, most notably affecting the cardiovascular, renal, cutaneous, and nervous systems either independently or in a combinatorial manner [2,3]. The manifestation and severity of FD depend on many factors, including the patient’s age of onset, sex, and specific genomic mutation, as there are over 1000 variants known currently [2,4]. FD produces a variety of clinical presentations, which can create a challenge in its prognosis and diagnosis as a whole.

Given its X-linked inheritance, FD was historically only screened and detected in men, with hemizygous men being the dominant patient population. However, in more recent years, increasing numbers of heterozygous women have been recognized as symptomatic FD patients, rather than asymptomatic carriers alone [5]. These female patients have unpredictable, broad severities in their disease phenotypes, ranging from complete absence of symptoms to life-threatening manifestations with multi-organ involvement [6]. It has been hypothesized that the large variability in FD traits among heterozygous women may be, in part, due to X-linked chromosomal inactivation (XCI), where one X chromosome is randomly silenced in each cell. If more cells in a specific organ express the X chromosome with the mutated GLA (galactosidase alpha) gene, known as skewed XCI, a woman may become symptomatic and warrant enzyme replacement therapy (ERT), the most common treatment of FD today [7-9]. As mentioned earlier, the vast number of genetic variants linked to FD may also contribute to the phenotypic variability seen in heterozygous female patients. Many of these variants are associated with missense and nonsense mutations, partial deletions and insertions, frameshift errors, and changes in splicing in the gene encoding alpha-galactosidase A [4,10]. Thus, the genomic location of the variant and the extent to which it disrupts protein structure or function may also contribute to the observed phenotypic heterogeneity discussed. 

FD has been commonly reported for its renal and cardiovascular implications, but it has a wide scope of clinical presentations based on the organ system(s) affected. Common notable symptoms, which can be combinatorial or isolated, include acroparesthesia, left ventricular hypertrophy, abdominal pain, cornea verticillata, chronic kidney disease, and obstructive lung disease, among many others [6,8]. Out of all known symptoms, angiokeratoma development is a hallmark of FD. Angiokeratomas are small, dark red or purple skin lesions that appear in the groin, hip, or periumbilical areas due to the lysosomal accumulation of glycosphingolipids in vascular endothelial cells [2,11]. While glycosphingolipid accumulation in the skin is a known result of alpha-galactosidase A deficiency, extracellular lipid accumulation in other organ systems has, to our knowledge, rarely been reported [2,6]. Here, we report a unique case of a female patient with Fabry disease presenting with a lung nodule, without any other organ involvement noted. Given that the lung nodule appears to be associated with localized lipid accumulation, this case highlights the variability in FD presentation in female patients and potential mechanisms underlying nodule formation in FD patients as an area for further study.

## Case presentation

A 31-year-old woman from Morocco with a family history of a father and son with FD presented to the emergency department after two episodes of hemoptysis with white sputum production streaked with red blood. She had been experiencing a cough for two days prior to her presentation. She also reported right-sided chest discomfort and headaches with no fever, chills, dizziness, numbness, weakness, bowel issues, weight loss, or abdominal pain. She had no smoking history and reported no alcohol or drug use. She stayed at home with no exposure to dust, chemicals, fumes, or smoke. The only medications she was taking were a daily multivitamin and ibuprofen as needed. Her surgical history was notable for arteriovenous (AV) fistula repair, cesarean section, and breast surgery.

**Figure 1:**
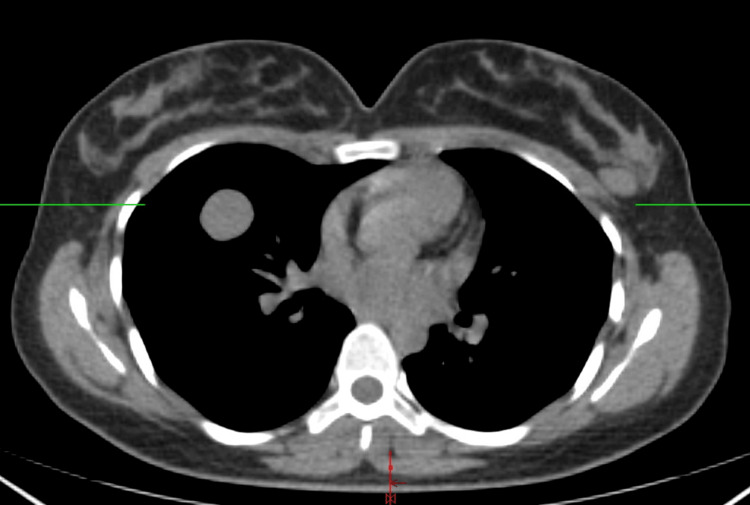
CT scan CT scan of the chest showing a 2.5 cm x 2.6 cm x 2.4 cm round nodule in the right middle lobe with suspected calcification.

Complete blood count (CBC) and comprehensive metabolic panel (CMP) were all within the normal range. A CT scan of the chest was performed and demonstrated a 2.5 cm x 2.6 cm x 2.4 cm round nodule in the right middle lobe with suspected calcification (Figure 1). There were internal areas of high density mostly near the periphery of the nodule, and the original differential was hamartoma, previous granulomatous infection, or carcinoid tumor. The patient was then discharged from the hospital with instructions to follow up in the pulmonary clinic as an outpatient.

The patient presented to the pulmonary clinic five days later, reporting no hemoptysis since discharge. A PET scan was recommended to further identify the lesion. The mass was found to have significantly increased fluorodeoxyglucose (FDG) metabolism with a standardized uptake value maximum of 12.2, concerning at the time for malignancy versus atypical infection. Due to its central location, a diagnostic bronchoscopy and biopsy were recommended.

**Figure 2:**
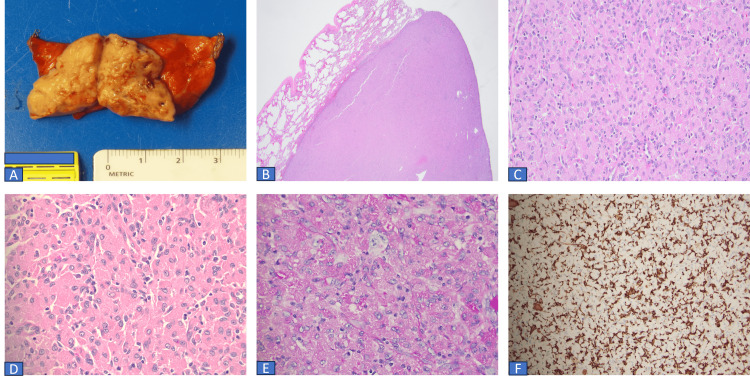
Gross and histopathologic findings A. The wedge resection showed a gray-tan bosselated mass measuring 4.0 cm in greatest dimension. B. The histologic sections showed a well-circumscribed mass involving the lung parenchyma (H&E 20 x). C. The lesion was composed of sheets of polygonal cells with abundant, eosinophilic, coarsely granular cytoplasm (H&E 100 x). D. The lesion’s cells showed no mitoses or necrosis (H&E 200 x). E. PAS stain highlights the foamy cytoplasm (PAS 200x). F. CD163 highlights the sheets of macrophages (IHC 100 x). H&E: hematoxylin and eosin; IHC: immunohistochemistry; PAS: periodic acid–Schiff

Roughly four weeks later, the patient underwent bronchoscopy with complete removal of the lesion (Figure 2A). Upon further pathological examination, the mass was described as well-defined and composed of predominantly polygonal cells with abundant, coarsely granular cytoplasm. There were no mitoses, no necrosis, and no vascular invasion. It appeared to be mimicking a granular cell tumor due to lipid accumulation in the cells. The sample was then subjected to multiple immunological and special stains (Figures 2B-2F). One notable finding was the periodic acid-Schiff (PAS)-D/PAS stain, which demonstrated foamy PAS-positive granules, potentially indicative of a lipid accumulation disorder. Results also included a negative S100 stain, indicating that a granular tumor was unlikely. The negative S100 and positive PAS stain argued against a pulmonary hamartomatous etiology of the lesion. Negative Grocott's methenamine silver (GMS) and acid-fast bacillus (AFB) staining, as well as negative fungal cultures, effectively ruled out a fungal or mycobacterial etiology. Finally, while not definitive, AE1/AE3 negativity made a carcinoid tumor less likely. At this point, it was suggested that the nodule appeared to be consistent with a possible atypical manifestation of FD, and the patient was recommended for genetic testing and further workup.

Upon genetic testing, the patient was found to have a *GLA* c.988C>G, p. Q330E novel variant that was classified as likely pathogenic. This was the same variant as her son, who was reported to have FD on his newborn screening. Testing three years prior, the patient identified low-normal alpha-galactosidase levels in plasma (7.4 nmol/hr/mL; reference range 6.6-21.4 nmol/hr/mL) with normal leukocyte alpha-galactosidase levels (34.0 nmol/hr/mg; reference range 12-99 nmol/hr/mg). Lyso-Gb3 levels were not tested in the patient. At her follow-up genetic counseling appointment, the patient reported no other signs or symptoms of FD. She received an echocardiogram that demonstrated normal heart function and showed no significant renal issues. The patient was ultimately recommended for biannual follow-up to check for further signs and symptoms of FD development.

## Discussion

This case highlights a previously undescribed, non-classical presentation of FD in a young, heterozygous female patient, who manifested with a solitary pulmonary nodule in the absence of typical systemic features. FD is known for its phenotypic heterogeneity, especially in female patients, where disease expression is strongly influenced by patterns of X-chromosome inactivation (XCI). Although XCI skewness was not assessed in this patient, it is still one possible explanation for the novel phenotype reported in this case. Skewed XCI can result in substantial variability of FD presentation, ranging from asymptomatic carriers to women with severe multi-organ involvement. In this patient, the finding of a lung nodule as the sole presentation underscores both the complexity of FD and the need for heightened clinical suspicion in atypical cases [10,12].

Additionally, the patient was found to harbor the *GLA *c.988C>G, p.Q330E variant, classified as likely pathogenic. While data on this specific mutation are scarce, variants at the same amino acid position have been reported in association with gonadal mosaicism, further emphasizing the genetic diversity and complexity of FD [8,13]. Furthermore, the p.Q330E variant was identified in the ClinVar database as potentially altering intron or non-coding sequences, both of which may lead to mishaps in splicing [14]. This finding highlights the need for a better understanding of the vast genetic variants associated with FD.

The absence of renal, cardiac, or dermatologic manifestations, despite pathological and genetic determination of FD, suggests that localized glycosphingolipid accumulation could possibly manifest without the systemic burden typically associated with disease progression. This observation raises the possibility that certain *GLA* variants may predispose to tissue-specific deposition, a hypothesis that warrants further investigation [11,15]. No other known variant of FD has been associated with gonadal mosaicism, thus underscoring the potential rarity of this variant not only in its unique phenotype but in its transmission as well [8,13].

This case also features a novel phenotype of FD. The patient displayed a nodule in the middle lobe of her right lung; this was attributed to localized accumulation of Gb3 and lyso-Gb3 resulting from impaired *GLA* function. While lung nodule formation has not previously been reported as a clinical manifestation of FD, accumulation of Gb3 and lyso-Gb3 has been associated with promoting a pro-inflammatory, proliferative microenvironment [10,16,17]. In fact, one study found that FD patients are at a greater risk of developing benign and malignant tumors (17% risk) compared to the Italian general population (5.5% risk) [17]. While our patient’s nodule was not clearly defined as a tumor, this finding lends credence to the idea that cellular proliferations can be more common in patients with FD. One possible mechanism that may drive this proliferation is oxidative stress and DNA damage by Gb3 accumulation, which promote reactive oxygen species production, DNA instability, and mutagenesis [16]. Another possible mechanism is that chronic inflammation due to glycosphingolipid accumulation may reduce antioxidant defenses and enhance inflammatory cytokine activity, creating a milieu favorable to cellular growth and replication [16]. Thus, while Gb3 accumulation associated with FD may influence pathways involved in cellular proliferation, its role in pulmonary nodule formation still remains undefined. Notably, pulmonary manifestations of FD are typically limited to obstructive lung disease and small airway involvement, making this case of a well-circumscribed nodule particularly novel [3,6,18].

One unresolved aspect of this presentation is the elevated FDG uptake found on PET-CT. While atypical infection and malignancy were effectively ruled out by surgical pathology, as mentioned earlier, this leaves an unclear etiology for the significantly increased uptake. Research in this field is limited; however, glycosphingolipid accumulation in FD triggering inflammation could provide a biologic rationale for this finding. One study looking at FD cardiomyopathy identified focal 18F-FDG uptake in seven out of 20 tested asymptomatic females [19], suggesting a potential relationship between glycosphingolipid accumulation and inflammation [20]. While there are plausible etiologies that may explain the FDG elevation observed, the finding remains incompletely resolved and has not been causally linked to the novel presentation of FD seen in this case. 

The final limitation of this report is the lack of explicit immunohistochemistry for FD. The lack of Sudan Black staining, Gb3 staining, and electron microscopy makes it difficult to definitively diagnose it as a manifestation of FD. However, family history and genetic testing confirm this as a definite possibility for this nodule. The accumulation of PAS/PAS-D positive granules and lack of positive staining for other etiologies also rule out other etiologies and argue in favor of the ultimate diagnosis of an FD manifestation. 

Overall, it is clear that there are still unknown aspects of the novel presentation of FD reported in this case. Further studies should be conducted to understand the mechanisms underlying extracellular lipid accumulation linked to FD, as well as the specific genetic variant, c.988C>G, p.Q330E, identified in this patient. It is especially important to understand the time course of nodule formation as well as why lipid accumulation in this patient was only localized to the lung. These findings will undoubtedly improve early prognosis and diagnosis of FD, given its wide-ranging presentations in heterozygous females.

## Conclusions

This report adds to the growing body of evidence that FD exhibits greater clinical diversity than traditionally recognized. Importantly, this case illustrates a unique, probable manifestation of FD not previously described in the literature: a previously asymptomatic young woman presenting with a lung nodule as the first clinical expression of disease. FD should therefore be considered in the differential diagnosis of lung nodules in patients with a known family history. Early recognition of such atypical presentations is essential, not only for timely diagnosis but also for appropriate genetic counseling, risk stratification, and long-term monitoring. Furthermore, this case highlights the need for broader genotype-phenotype correlation studies, especially in females, to better define the spectrum of clinical outcomes associated with specific *GLA* variants. Accurate diagnosis and treatment of FD remain critical for effective follow-up and long-term management. Ultimately, this case emphasizes the unusual manifestations that FD may present with and provides valuable insight for clinicians evaluating unexplained lung lesions in affected patients.
